# The NuRD nucleosome remodelling complex and NHK-1 kinase are required for chromosome condensation in oocytes

**DOI:** 10.1242/jcs.158477

**Published:** 2015-02-01

**Authors:** Elvira Nikalayevich, Hiroyuki Ohkura

**Affiliations:** Wellcome Trust Centre for Cell Biology, The University of Edinburgh, Mayfield Road, Edinburgh EH9 3JR, UK

**Keywords:** Chromosome, Condensation, *Drosophila*, Oocyte, Meiosis

## Abstract

Chromosome condensation during cell division is one of the most dramatic events in the cell cycle. Condensin and topoisomerase II are the most studied factors in chromosome condensation. However, their inactivation leads to only mild defects and little is known about the roles of other factors. Here, we took advantage of *Drosophila* oocytes to elucidate the roles of potential condensation factors by performing RNA interference (RNAi). Consistent with previous studies, depletion of condensin I subunits or topoisomerase II in oocytes only mildly affected chromosome condensation. In contrast, we found severe undercondensation of chromosomes after depletion of the Mi-2-containing NuRD nucleosome remodelling complex or the protein kinase NHK-1 (also known as Ballchen in *Drosophila*). The further phenotypic analysis suggests that Mi-2 and NHK-1 are involved in different pathways of chromosome condensation. We show that the main role of NHK-1 in chromosome condensation is to phosphorylate Barrier-to-autointegration factor (BAF) and suppress its activity in linking chromosomes to nuclear envelope proteins. We further show that NHK-1 is important for chromosome condensation during mitosis as well as in oocytes.

## INTRODUCTION

During cell division, chromosomes undergo morphological changes from a cloud-like interphase morphology into rod-like structures. This transformation is referred to as chromosome condensation. Chromosome condensation is important for faithful chromosome segregation during cell division. The organisation of condensed metaphase chromosomes has been a focus of debate for a long time, and various models have been proposed (Luger et al., 2012). One model is that there is a hierarchical organisation, starting from nucleosomes folded into a 30-nm fibre, which form larger and larger loops (Belmont et al., 1987; Sedat and Manuelidis, 1978). Another long-standing, and not mutually exclusive, model is that there is a chromosome scaffold, which has been observed after removal of DNA and most of the chromosome proteins from the metaphase chromosomes (Marsden and Laemmli, 1979). However, the existence and the biological role of this scaffold are subjects of continuous discussion. The most recently proposed model is a polymer model based on data from a chromosome conformation capture method (Naumova et al., 2013). This proposes that there is a compressed linear array of loops without hierarchical organisation.

Among thousands of proteins found in metaphase chromosomes, condensin complexes and topoisomerase II have been studied most extensively for their involvement in chromosome condensation during cell division. The condensin complex was originally found as the main chromosome condensation factor in *Xenopus* extract (Hirano and Mitchison, 1994; Hirano et al., 1997). The involvement of condensin complexes in this process has been demonstrated in many systems (Hudson et al., 2003; Ono et al., 2003; Hagstrom et al., 2002; Steffensen et al., 2001; Sutani et al., 1999). Higher eukaryotes have two condensin complexes, condensin I and II (Hirano, 2012). The two complexes appear to have different localisations and functions. The exact molecular mechanism by which condensin functions has not been established, but it has an ability to positively supercoil DNA (Kimura and Hirano, 1997).

It has been demonstrated in several systems that topoisomerase II is required for chromosome structure as well as correct chromosome segregation in mitosis and meiosis (Uemura et al., 1987; Spence et al., 2007; Adachi et al., 1991; Li et al., 2013). Topoisomerase II is present on chromosomes in mitosis and meiosis (Earnshaw and Heck, 1985; Maeshima and Laemmli, 2003; Lee et al., 2011) and is also enriched on centromeres and pericentromeric regions during meiosis (Lee et al., 2011). Topoisomerase II decatenates supercoiled DNA by introducing temporary double-strand DNA breaks, and it has been suggested and demonstrated that topoisomerase II acts in opposition to condensin and KIF4A (Baxter and Aragón, 2012; Samejima et al., 2012). Both condensin and topoisomerase II are required for the correct chromatin structure of the centromere (Hirano, 2012; Vagnarelli, 2013).

Despite extensive research on the roles of condensin and topoisomerase II in chromosome condensation, some evidence casts doubts on whether these proteins are the only major factors involved in chromosome condensation. In some systems, chromosomes are still able to condense, with various abnormalities, after depletion of condensin subunits (Coelho et al., 2003; Hirota et al., 2004; Hudson et al., 2003; Ribeiro et al., 2009) or topoisomerase II (Carpenter and Porter, 2004; Chang et al., 2003; Sakaguchi and Kikuchi, 2004). Depletion of condensin does not prevent condensation of chromosomes until the initiation of anaphase, but causes chromosomes to decondense prematurely during anaphase (Vagnarelli et al., 2006). This has led to a proposal that there is a ‘regulator of chromosome architecture’ (RCA), an as yet unidentified factor, which acts redundantly with condensin to condense metaphase chromosomes (Vagnarelli et al., 2006).

Evidence suggests that there are crucial chromosome condensation factors other than condensin and topoisomerase II. Recently, attempts have been made to find new chromosome condensation factors. For example, a chromosome condensation assay allowed high-throughput analysis of genes required for chromosome condensation in fission yeast (Petrova et al., 2013). In that study, eight new conditional condensin alleles were discovered, together with a new role for DNA polymerase ε (pol ε) and F-box DNA helicase I in chromosome condensation (Petrova et al., 2013). In addition, a very recent study has identified mutations in several genes that cause chromosome segregation defects similar to those induced by depletion of condensin. Four out of five of these genes encode components of the nucleosome-remodelling complexes (Robellet et al., 2014).

In this report, we describe the first use of *Drosophila* oocytes to study chromosome condensation. RNA interference (RNAi)-mediated depletion of a set of chromosomal proteins revealed that depletion of the nucleosome-remodelling protein Mi-2 and the protein kinase NHK-1 (Nucleosomal histone kinase-1, also known as Ballchen in *Drosophila*) resulted in much more severe defects than depletion of well-known chromosome condensation factors. The condensation defects of Mi-2 and NHK-1 depletion were distinct from each other, suggesting that these proteins function in different pathways. We found that the main NHK-1 action in chromosome condensation is to suppress Barrier-to-autointergration factor (BAF) activity, which functions to link nuclear envelope proteins to chromosomes.

## RESULTS

### Identification of chromosome condensation factors revealed multiple pathways of condensation

Molecular mechanisms of chromosome condensation in cells remain poorly understood. This is partly because only a limited number of factors have been identified, and observed condensation defects are generally mild when factors are disrupted in mitotic cells (Hirota et al., 2004; Ribeiro et al., 2009; Carpenter and Porter, 2004; Sakaguchi and Kikuchi, 2004). We rationalised that the use of *Drosophila* oocytes might provide a unique insight into chromosome condensation. For example, as the volume of *Drosophila* oocytes dramatically increases after the last mitotic division, effective depletion of even stable proteins can be achieved by RNAi.

**Fig. 1.**
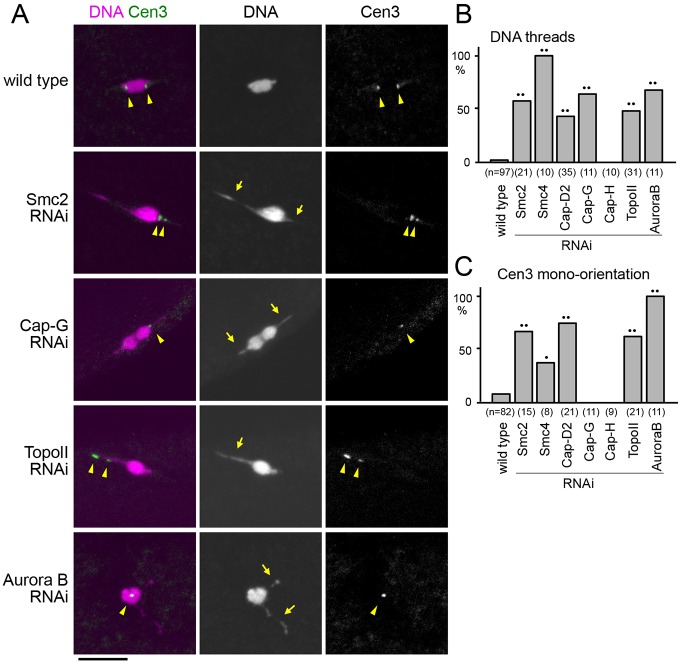
**RNAi of various chromosomal proteins causes different chromosome condensation defects in mature oocytes.** (A) Chromosome morphology (DNA; DAPI staining) and the positions of centromere 3 (Cen3; Dodeca satellite) in mature oocytes expressing the indicated shRNA. Arrows and arrowheads indicate thin DNA threads and Cen3 signals, respectively. Scale bar: 10 µm. (B) The frequencies of thin DNA thread occurrence in wild-type oocytes or oocytes expressing shRNA for *Smc2*, *Smc4*, *CapD-2*, *Cap-G*, *Cap-H*, *Topoisomerase II* (TopoII) or *Aurora B*. (C) The frequencies of mono-orientated centromere 3, in wild-type oocytes or oocytes expressing shRNA for *Smc2*, *Smc4*, *Cap-D2*, *Cap-G*, *Cap-H, Topoisomerase II* or *Aurora B*. Cen3 mono-orientation was defined as two Cen3 signals located on the same side of the chromosome mass or one unseparated Cen3 signal. ^•^*P*<0.05; ^••^*P*<0.01 compared with wild type.

To identify chromosome condensation factors in *Drosophila* oocytes, we knocked down potential factors involved in this process in oocytes. As the first step, categories of proteins known to be involved in chromosome-related functions were selected, including chromatin-modifying enzymes, nucleosome remodelling factors, chromatin insulators and helicases. Among these, transgenic lines expressing short hairpin RNA (shRNA) constructs were available for 51 genes (supplementary material Table S1). The expression of shRNA was driven in the female germline after the completion of mitotic divisions and recombination using GAL4 driven by the maternal *α-tubulin67A* promoter (Radford et al., 2012). A total of 30 sterile or poorly fertile lines were further selected for cytological analysis. DNA staining of mature oocytes, which naturally arrest in metaphase I (Ashburner et al., 2005), revealed that RNAi-mediated depletion of eight genes gave chromosome condensation defects (Fig. 1). A reduction in the amount of the corresponding mRNA was confirmed by quantitative RT-PCR (supplementary material Fig. S1A). Possibilities of off-target effects were subsequently excluded for all hits except for topoisomerase II and Aurora B by testing non-overlapping shRNA for the same gene, shRNA targeting genes for other subunits of the same complex and/or perturbation of known substrates (see below).

These positive hits could be broadly grouped into two categories. The first category consists of well-known condensation factors, including condensin I subunits, Topoisomerase II and Aurora B, which showed mild condensation defects after RNAi (Fig. 1A). In oocytes expressing shRNA for condensin I subunits and topoisomerase II, the majority of chromosomes were compacted into the main chromosome mass but thin threads often emanated from the main mass (Fig. 1A,B). Fluorescent *in situ* hybridisation (FISH) probing a peri-centromeric satellite on the third chromosome showed that the satellites were often found in the thin threads (Fig. 1A), suggesting that centromeric and peri-centromeric regions were the main regions of undercondensation. In addition, these peri-centromeric satellites were often found at the same side of the chromosome mass (Fig. 1A,C), indicating mis-orientation of homologous centromeres. Depletion of four out of five condensin I subunits showed a similar chromosome defect but with various frequencies (Fig. 1), suggesting that depletion of target proteins was achieved by RNAi at a high frequency but that the levels of depletion might vary. Most oocytes expressing Aurora B shRNA showed thin chromatin threads connected to condensed chromosomes (Fig. 1). As Aurora B RNAi resulted in severely compromised microtubule assembly (Radford et al., 2012; supplementary material Fig. S1B), the thin chromatin threads are not due to pulling forces on the chromosomes.

**Fig. 2.**
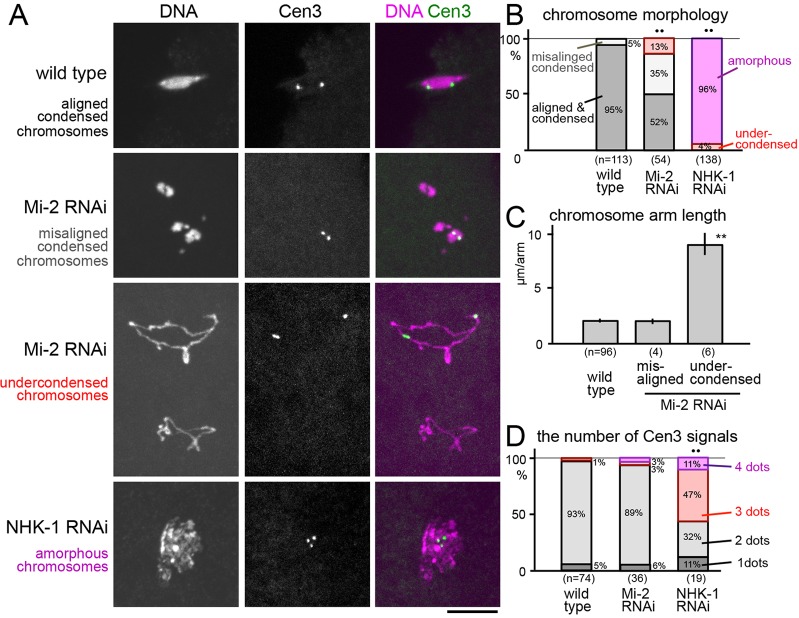
***Mi-2* and *NHK-1* RNAi lead to chromosome undercondensation in mature oocytes.** (A) Chromosome morphology (DNA; DAPI staining) and the positions of centromere 3 (Cen3; Dodeca satellite) in mature oocytes expressing shRNA for *Mi-2* or *NHK-1*. Representative images show aligned condensed chromosomes in wild-type oocytes, misaligned condensed chromosomes and undercondensed chromosomes in *Mi-2* RNAi oocytes, and amorphous chromosomes in *NHK-1 RNAi* oocytes are shown. Scale bar: 10 µm. (B) Quantification of chromosome morphology in wild-type oocytes or oocytes expressing shRNA for *Mi-2* or *NHK-1*. ‘Undercondensed’ chromosomes in *NHK-1* RNAi oocytes were irregularly undercondensed chromosomes which are distinct from those seen in Mi-2 *RNAi* oocytes. ^••^*P*<0.01 in the frequency of undercondensed or amorphous chromosomes compared with wild type. (C) The estimated length of chromosome arms (assuming ten equal sized chromosome arms in diploid oocytes) in wild-type oocytes or *Mi-2* RNAi oocytes containing misaligned or undercondensed chromosomes. Results are mean±s.e.m. **P<0.01 compared with wild type. (D) The number of Cen3 signals in wild-type oocytes, or oocytes expressing shRNA for *Mi-2* or *NHK-1*. *n* = 74, 36 and 19, respectively. ^••^*P*<0.01 in the frequency of three or four dots compared with wild type.

The second category consists of the nucleosome remodelling factor Mi-2 and the conserved kinase NHK-1, for which RNAi-mediated depletion caused severe chromosome condensation defects in oocytes (Fig. 2), in comparison to the well-known chromosome condensation factors mentioned above. This demonstrated that the *Drosophila* oocyte combined with RNAi is an effective system to identify crucial chromosome condensation factors.

### *Mi-2* and *NHK-1* RNAi showed distinct chromosome condensation defects

We focused our analysis on chromosome condensation defects in the *Mi-2* and *NHK-1* RNAi condition in oocytes, as previous studies of these two proteins had not revealed their roles in promoting condensation of metaphase chromosomes. A study using overexpression of wild-type and dominant-negative Mi-2 has suggested that Mi-2 induces chromosome decondensation in polytene and mitotic cells (Fasulo et al., 2012). Hypomorphic female sterile *nhk-1* mutants show fully condensed metaphase chromosomes in mature oocytes (Cullen et al., 2005; Ivanovska et al., 2005). Furthermore RNAi or mutations of the *NHK-1* orthologues has not revealed chromosome undercondensation in mitosis (Cullen et al., 2005; Ivanovska et al., 2005; Gorjánácz et al., 2007; Molitor and Traktman, 2014). The apparent dissimilarities between previous studies and ours might be due to differences in methodologies and/or systems.

To further characterise chromosome condensation defects, the chromosome morphology of mature oocytes expressing shRNA for *Mi-2* or *NHK-1* was examined in detail. Oocytes expressing *Mi-2* shRNA showed a low, but significant, frequency (13%) of undercondensation of chromosomes (Fig. 2A–C). In addition, *Mi-2* shRNA induced chromosome misalignment (35%) without clear condensation defects (Fig. 2A). These undercondensed chromosomes appeared to be elongated by more than fourfold in comparison with condensed chromosomes in wild-type or Mi-2 shRNA conditions (Fig. 2C). In contrast, oocytes expressing *NHK-1* shRNA did not have normally condensed chromosomes, and nearly all of them had amorphous DNA strings without recognisable individual chromosomes (Fig. 2A,B).

To further investigate the chromosome organisation, FISH was carried out using a peri-centromeric satellite on the third chromosome as a probe. Wild-type oocytes had two dots, which represent a pair of homologous centromeres on the edges of the chromosome mass, and each dot comprises the signals for closely located sister centromeres (Fig. 2A,D). In *Mi-2* RNAi oocytes, two dots were found on undercondensed chromosomes highlighting a bivalent structure with chiasmata (Fig. 2A,D). In contrast, most (58%) of the *NHK-1* RNAi oocytes contained three or four separate dots (Fig. 2A,D), suggesting compromised attachment between sister chromatids.

### Mi-2 and NHK-1 affect chromosome organisation at different stages

**Fig. 3.**
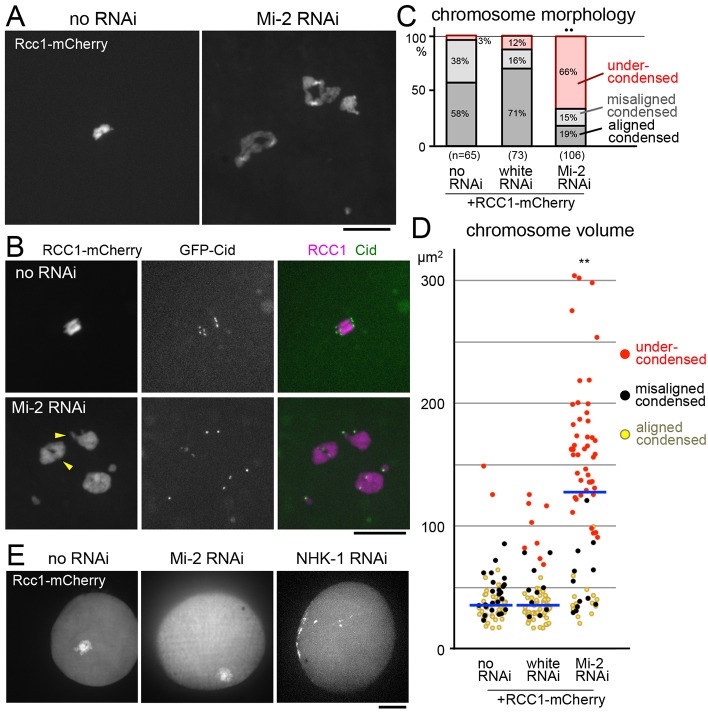
**Live imaging reveals that NHK-1 and Mi-2 function at different stages.** (A) Chromosome undercondensation in mature oocytes expressing Rcc1–mCherry alone (no RNAi) or Rcc1–mCherry and shRNA for *Mi-2*. Scale bar: 10 µm. (B) Chromosome morphology and centromere (Cid) positions in mature oocytes expressing Rcc1–mCherry, GFP–Cid or *Mi-2* shRNA. Chromosomes were narrower in centromeric bad pericentromeric regions (arrowheads). Scale bar: 10 µm. (C) Quantification of chromosome morphology in mature oocytes expressing Rcc1–mCherry alone (no RNAi), or Rcc1–mCherry and shRNA for *white* (an unrelated gene as a control) or *Mi-2*. ^••^*P*<0.01 in the frequency of undercondensed chromosomes compared with wild type. (D) The volume occupied by chromosomes in oocytes expressing Rcc1–mCherry alone (no RNAi) or Rcc1–mCherry and shRNA for *white* (an unrelated gene as a control) or *Mi-2*. The blue horizontal lines represent the median. **P<0.01 in the chromosome volume compared with wild type. (E) The structure of the karyosome (clustered chromosomes) in late prophase I oocytes expressing Rcc1–mCherry alone (no RNAi), or Rcc1–mCherry and shRNA for *Mi-2* or *NHK-1*. The karyosome structure in late prophase I was disrupted in NHK-1 RNAi oocytes, but not Mi-2 RNAi oocytes. Scale bar: 10 µm.

Next, the chromosome morphology was examined in live oocytes using maternally expressed Regulator of chromosome condensation 1 (RCC1) tagged with mCherry as a chromosome marker (Fig. 3). This expression of RCC1–mCherry alone in a wild-type background induced chromosome undercondensation only in 3% of oocytes (Fig. 3C). When *Mi-2* shRNA was induced together with RCC1–mCherry, a high frequency (66%) of chromosome undercondensation was observed in mature live oocytes (Fig. 3A,C), and the chromosomes were not only longer, but also wider, occasionally with some more condensed regions (Fig. 3A). DAPI staining of fixed oocytes indicated that expression of Rcc1–mCherry indeed enhanced the *Mi-2* RNAi condensation defect (supplementary material Fig. S2). GFP-tagged Cid (the Cenp-A orthologue), which highlights centromeres (Schuh et al., 2007), was often associated with narrower regions of chromosomes (Fig. 3B). This suggests that *Mi-2* RNAi has a different effect on chromosome arms to its effect on centromeric and peri-centromeric regions. In *NHK-1* RNAi oocytes expressing Rcc1–mCherry, we could not reliably recognise amorphous chromosomes over the background signal. To quantify the degree of chromosome condensation, the volume occupied by all chromosomes (the Rcc1–mCherry signal) was measured in live oocytes (Fig. 3D). We found that the volume of chromosomes increased after *Mi-2* depletion. The median volume of all chromosomes was increased fourfold.

To gain an insight into when these two proteins function, we observed the chromosome organisation in late prophase I. Unlike immunostaining, the live imaging procedure retains the dorsal appendages of oocytes, which can be used to determine the oocyte stage. Maturing oocytes at stage 12 or 13 were selected by their dorsal appendage morphology and chromosomes were observed before nuclear envelope breakdown. In wild-type late prophase I oocytes, meiotic chromosomes clustered together to form a compact body called the karyosome within the enlarged nucleus (*n* = 13; Fig. 3E). The karyosome organisation of *Mi-2* RNAi oocytes appeared normal before nuclear envelope breakdown (*n* = 17; Fig. 3E). In contrast, in *nhk-1* RNAi oocytes, even before nuclear envelope breakdown the karyosome was severely disrupted (*n* = 4) or invisible against nucleoplasmic background, probably owing to the highly diffused morphology of the chromosomes (*n* = 7; Fig. 3E). This defect in late prophase I is consistent with previous observations of disrupted karyosomes at earlier stages in hypomorphic *nhk-1* mutants, although metaphase chromosomes are fully condensed in mature oocytes carrying these alleles (Cullen et al., 2005; Ivanovska et al., 2005). The differences in chromosome morphology in prophase I and metaphase I suggest that NHK-1 and Mi-2 are involved in distinct pathways from each other in chromosome condensation.

### Knockdown of Mi-2 and NHK-1 disrupts spindle formation

To test whether Mi-2 or NHK-1 are required for spindle formation, RNAi oocytes were immunostained for α-tubulin. A Mi-2 orthologue, CHD4, has been recently shown to bind microtubules and to be required for full spindle microtubule assembly and spindle bipolarity in *Xenopus* egg extract and cultured cells from humans and *Drosophila* (Yokoyama et al., 2013). In *Mi-2* RNAi oocytes, about a half of the oocytes had abnormal spindles (supplementary material Fig. S3). The abnormalities include unfocused poles, multiple spindles and microtubule asters around chromosomes. In hypomorphic *nhk-1* mutants, it was previously shown that spindle microtubule assembly is normal, although multiple spindles are often formed. In *NHK-1* RNAi oocytes, spindle microtubule assembly was severely compromised (supplementary material Fig. S3). It remains to be determined whether the spindle assembly defects in these RNAi conditions are directly caused by a loss of NHK-1 or Mi-2 activity, or are a secondary consequence of the chromosome defects.

### The NuRD complex containing Mi-2 is responsible for chromosome condensation

In *Drosophila*, Mi-2 is a subunit of two separate protein complexes, the NuRD complex and the MEC complex. The *Drosophila* NuRD complex consists of Mi-2, MTA1-like, MBD-like, Caf1, Rpd3 and p66 (also known as Simjang), whereas the MEC complex consists of Mi-2 and MEP-1 (Kunert et al., 2009). To determine which complex promotes condensation, shRNAs corresponding to specific subunits were expressed in oocytes expressing RCC1–mCherry. RNAi of *MTA1-like*, *MBD-like* and *Rpd3* showed condensation defects similar to *Mi-2* RNAi, whereas *MEP-1* RNAi did not show clear defects (supplementary material Fig. S4). As RNAi of 4 subunits of the NuRD complex showed similar condensation defects, we conclude that the NuRD complex is responsible for promoting chromosome condensation.

### BAF phosphorylation by NHK-1 is required for chromosome condensation

**Fig. 4.**
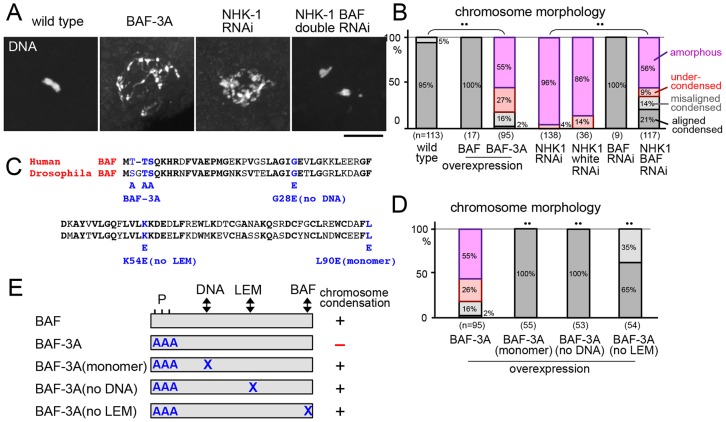
**NHK-1 suppresses the activity of BAF in linking nuclear envelope proteins to DNA.** (A) DNA staining of mature oocytes expressing non-phosphorylatable BAF-3A, shRNA for *NHK-1*, or shRNAs for both *NHK-1* and *BAF*. The condensation defect caused by *NHK-1* RNAi is phenocopied by expression of non-phosphorylatable BAF and is rescued by *BAF* RNAi. Scale bar: 10 µm. (B) Chromosome morphology in wild-type oocytes, oocytes overexpressing BAF or BAF-3A, or oocytes expressing shRNA for *NHK-1*, *NHK-1* and *white*, *BAF*, or *NHK-1* and *BAF*. ^••^*P*<0.01 between the indicated pairs in the frequency of undercondensed or amorphous chromosomes. (C) The sequence of human and *Drosophila* BAF, together with amino acid mutations shown or predicted to disrupt interactions with DNA (no DNA), the LEM domain (no LEM) and BAF itself (monomer) in human BAF and the equivalent mutations in *Drosophila* BAF. (D) Chromosome morphology of mature oocytes expressing BAF-3A and BAF-3A variant containing each mutation. These interactions are required for non-phosphorylatable BAF to prevent chromosome condensation in oocytes. ^••^*P*<0.01 in the frequency of undercondensed or amorphous chromosomes compared with BAF-3A expression. (E) Diagram of BAF variants and their overexpression effects on chromosome condensation. + and − indicate proper condensation and severe undercondensation, respectively.

Next, we investigated the molecular mechanism of NHK-1 function in chromosome condensation. One known NHK-1 substrate is BAF, a linker between DNA and LEM-domain-containing nuclear envelope proteins (Segura-Totten and Wilson, 2004; Furukawa, 1999). The phosphorylation of BAF has been previously shown to suppress the BAF interactions with both DNA and LEM-domain-containing nuclear envelope proteins (Nichols et al., 2006). To identify the role of BAF phosphorylation in chromosome condensation, non-phosphorylatable BAF (BAF-3A; Lancaster et al., 2007) was expressed in otherwise wild-type oocytes. Most oocytes expressing BAF-3A showed a severe chromosome condensation defect similar to the one induced by *NHK-1* RNAi, whereas expression of wild-type BAF did not affect chromosome condensation (Fig. 4A,B).

To determine whether and how much the condensation function of NHK-1 is mediated by BAF, we have tested whether depletion of BAF can rescue the condensation defect caused by *NHK-1* RNAi. Double RNAi of *BAF* and *NHK-1* was compared with single *NHK-1* RNAi, single *BAF* RNAi, and double RNAi of *NHK-1* and an unrelated gene (*white*) as a control. Double RNAi of BAF and NHK-1 led to fully condensed chromosomes (40%), which were not seen in single NHK-1 RNAi. Single BAF RNAi did not show any defects, and double RNAi of NHK-1 and the unrelated gene did not change the severe condensation defect of single NHK-1 RNAi (Fig. 4A,B). This rescue demonstrated that suppression of BAF activity is the main function of NHK-1 in chromosome condensation in oocytes.

BAF is a small protein which forms a homodimer, and directly binds to DNA and LEM domains of multiple inner nuclear membrane proteins (Zheng et al., 2000; Furukawa, 1999; Shumaker et al., 2001). Residues involved in these interactions have been identified in other systems (Segura-Totten et al., 2002; Cai et al., 1998). To test which of these protein interactions are essential for the BAF-3A effect, crucial residues were mutated in BAF-3A, and mutant BAF-3A was expressed in otherwise wild-type oocytes. We found that mutations disrupting binding to either DNA (‘no DNA’), LEM (‘no LEM’) or to BAF itself (‘monomer’) abolished the condensation defects caused by BAF-3A expression (Fig. 4C–E). This indicates that BAF without phosphorylation prevents chromosome condensation by linking LEM-containing inner nuclear envelope proteins to DNA.

### Involvement of NHK-1 in chromosome condensation in mitosis

Finally, we addressed the question of whether the role of NHK-1 in chromosome condensation is specific to oocytes. For example, it is possible that the sole role of NHK-1 is to form the proper karyosome, which is specific to the oocyte nucleus, and that the chromosome condensation defect seen after *NHK-1* RNAi is simply a secondary consequence of the karyosome defect in prophase I.

**Fig. 5.**
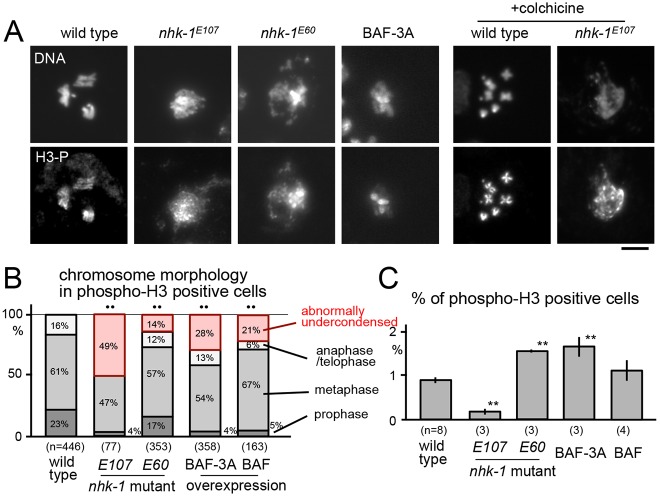
**NHK-1 is important for chromosome condensation in mitosis.** (A) Mitotic figures positive for phospho-H3 (at S10) in CNSs from wild-type larvae, wild-type larvae overexpressing BAF-3A or larvae that had one of two lethal mutant alleles of *nhk-1*. The null allele *E107* was examined in homozygotes and the hypomorphic allele *E60* was examined over a deficiency uncovering *nhk-1*. +colchicine indicates mitotic figures from the larval central nervous systems incubated with colchicine. Scale bar: 10 µm. (B) Frequencies of chromosome morphology classes in phospho-H3-positive cells in larval CNSs from wild type, *nhk-1^E60^*, *nhk-1^E107^* or flies overexpressing BAF-3A or BAF. **P<0.01 in the frequency of abnormally undercondensed chromosomes compared with wild type. (C) The frequency of mitotic (phospho-H3 positive) cells. Results are mean±s.e.m. derived from biological triplicates. **P<0.01 compared with wild type.

To address this question, we examined the chromosome morphology of *nhk-1* mutants during mitosis. Central nervous systems from third-instar larvae were fixed, squashed and stained for DNA and histone 3 phosphorylated at S10 (phospho-H3), a mitosis-specific marker. Two lethal *nhk-1* mutants, null (*E107*) and hypomorphic (*E60*) alleles (Cullen et al., 2005), were examined together with the wild type. In wild-type larvae, all mitotic (phospho-H3 positive) cells showed condensed chromosomes or were in telophase (Fig. 5). In contrast, in *nhk-1* mutants a significant proportion of phospho-H3 positive cells had parts of chromosomes that were abnormally undercondensed (49% in *E107* and 14% in *E60*; Fig. 5). The frequency of chromosome bridges or lagging chromosomes increased in *nhk-1^E60^* (10 out of 53 anaphases and telophases versus 1 out of 111 in wild type; *P*<0.01). These results support the involvement of NHK-1 in condensation of mitotic chromosomes, although we could not exclude the possibility that H3 phosphorylation was affected in the mutants or that these abnormal figures resulted from prolonged metaphase arrest.

To test whether this phenotype is mediated by BAF phosphorylation in mitosis, wild-type BAF and non-phosphorylatable BAF (BAF-3A) were overexpressed in otherwise wild-type flies (Fig. 5). We found that both showed abnormally undercondensed chromosomes in a significant proportion of phospho-H3-positive cells (28% in BAF-3A and 21% in BAF). This is in contrast to oocytes, in which only the non-phosphorylatable BAF prevented chromosome condensation. It is possible that overexpressed wild-type BAF might remain unphosphorylated in mitosis owing to limited NHK-1 activity. Nevertheless, our results suggest that the NHK-1 substrate BAF is involved in chromosome condensation also in mitosis. These results show that NHK-1 is important for chromosome condensation not only during meiosis in oocytes but also during mitosis in somatic cells.

## DISCUSSION

In this study, we used *Drosophila* oocytes as a new model system for chromosome condensation. Knockdown of potential chromosomal proteins or regulators by RNAi in oocytes has identified new factors promoting chromosome condensation (the NuRD complex and NHK-1) as well as known factors (condensin I, topoisomerase II and Aurora B). Depletion of the protein kinase NHK-1 and the NuRD nucleosome remodelling complex containing Mi-2 caused severe chromosome condensation defects that were distinct from each other. Further study revealed that BAF is the main substrate of NHK-1 for its chromosome condensation function and that NHK-1 promotes chromosome condensation by suppressing the linker activity of BAF between nuclear envelope proteins and DNA. Finally, we showed that NHK-1 is also important for chromosome condensation in mitosis.

This report is the first to use *Drosophila* oocytes to study chromosome condensation. We argue that the *Drosophila* oocyte combined with RNAi is an excellent system for research of chromosome condensation, which complements commonly used mitotic systems. Firstly, *Drosophila* oocytes grow enormously in volume between completion of pre-meiotic mitosis and recombination and chromosome condensation (Cummings and King, 1969). shRNA expression can be induced after the protein executes its role in the previous mitosis and/or recombination but prior to oocyte growth (Radford et al., 2012). Even if the target protein is stable, it becomes sufficiently diluted before chromosome condensation in oocytes. This is in contrast to mitotic cycles where cells only double in size between divisions. Secondly, *Drosophila* oocytes arrest in metaphase of the first meiotic division (Ashburner et al., 2005). This allows chromosome defects to be studied in the first division after the target protein is depleted, rather than as a mixture of defects accumulated through multiple divisions caused by a gradual decrease of the protein. Finally, as oocytes are large, the condensation state of chromosomes can be clearly observed without mechanical treatment such as squashing or spreading. Therefore, RNAi in *Drosophila* oocytes could be a powerful system to study chromosome condensation, although negative results should be interpreted with caution as they might be caused by insufficient depletion, genetic redundancy or cell-type-specific function.

Indeed, in this study, a small-scale survey of chromosomal proteins, new chromosome condensation factors were identified in addition to well-known ones, demonstrating the effectiveness of *Drosophila* oocytes as a research system. Well-known factors, including condensin I subunits, topoisomerase II and Aurora B, showed milder chromosome condensation defects. Knockdown of topoisomerase II or condensin I showed similar condensation defects, and appeared to affect mainly centromeric and/or pericentromeric regions. The previous reports in mitosis are consistent with our result, suggesting that these two factors are not the main condensation factors in mitosis or in meiosis (Hirota et al., 2004; Ribeiro et al., 2009; Carpenter and Porter, 2004; Sakaguchi and Kikuchi, 2004).

A previous study of Mi-2 in *Drosophila* suggested that it promotes decondensation of chromosomes because overexpression of wild-type Mi-2 results in chromosome decondensation in polytene or mitotic cells and overexpression of dominant-negative Mi-2 results in overcondensation (Fasulo et al., 2012). In our current study, Mi-2 RNAi in oocytes showed chromosome decondensation, whereas in our preliminary study in neuroblasts Mi-2 RNAi did not show chromosome decondensation. The difference from the previous study might be due to the method of disrupting the Mi-2 function or cell types used for the studies. We argue that the phenotype caused by RNAi in oocytes is a better reflection of the *in vivo* function. RNAi of other NuRD subunits indicated that the NuRD complex is important for chromosome condensation.

How does the NuRD complex promote chromosome condensation? It is possible that nucleosome remodelling is directly required during chromosome condensation. For example, proper positioning of nucleosomes might be important for full chromosome condensation. Indeed, other nucleosome remodelling complexes have been suggested to be involved in chromosome condensation in fission yeast (Robellet et al., 2014). Alternatively, histone deacetylase acivity of the NuRD complex might be important for chromosome condensation, as histone modifications are a major way to regulate chromosome structure (Wang and Higgins, 2013). We also cannot exclude the possibility that NuRD acts through transcription of other chromosome condensation factors, as it is known to regulate gene transcription (Ramírez and Hagman, 2009). Further studies using more sophisticated mutations would help to distinguish these possibilities.

We found that knockdown of NHK-1 resulted in severe chromosome condensation defects in nearly all oocytes. Previously, involvement of NHK-1 or its orthologues in metaphase chromosome condensation has not been reported, although overexpression of the human orthologue disrupts chromatin organisation in interphase (Kang et al., 2007). None of the three female sterile *nhk-1* mutants showed chromosome condensation defects in metaphase I in oocytes (Cullen et al., 2005; Ivanovska et al., 2005). This might be because the minimal NHK-1 activity required for producing viable adults is sufficient to allow chromosome condensation in oocytes. Female-germline-specific RNAi is likely to have achieved greater depletion of NHK-1 in oocytes. We showed that phosphorylation of BAF, thus inactivating its linking of DNA to LEM-domain-containing inner nuclear membrane proteins, is the major role of NHK-1 in chromosome condensation in oocytes. However, NHK-1 might regulate multiple pathways during condensation, for example, it has been shown that it is required for histone 2A phosphorylation and condensin recruitment in prophase I oocytes (Ivanovska et al., 2005).

A crucial question is whether the chromosome condensation defect is a direct consequence of NHK-1 loss or a secondary consequence of a karyosome defect in prophase I oocytes. Evidence indicates that the compact karyosome in the prophase I nucleus and chromosome condensation in metaphase I are at least partly independent. In female-sterile hypomophic *nhk-1* mutants, chromatin organisation in prophase I oocytes is defective, but metaphase I chromosomes are properly condensed in mature oocytes (Cullen et al., 2005; Ivanovska et al., 2005). By contrast, in Mi-2 RNAi oocytes, the karyosome is normal in prophase I, but chromosomes become undercondensed after nuclear envelope breakdown in some metaphase I oocytes. Furthermore, as chromosome condensation in mitosis is also defective in *nhk-1* mutants, the role for NHK-1 in chromosome condensation must be at least partly independent from meiosis-specific chromatin organisation. Therefore, release of LEM-containing nuclear envelope proteins from chromosomes might be a prerequisite for proper chromosome condensation.

In conclusion, our targeted survey using RNAi in *Drosophila* oocytes has already identified new factors required for chromosome condensation. Further analysis provided new insights into the molecular mechanism of condensation including the release of nuclear envelope proteins from chromosomes and nucleosome remodelling and/or histone deacetylation as essential steps for condensation. In future, a larger scale screen of putative chromosomal proteins might prove to be fruitful.

## METHODS AND MATERIALS

### Handling of *Drosophila melanogaster*

Standard methods of fly handling were used (Ashburner et al., 2005). Fly lines for RNAi used in this study were designed to express shRNA under the UASp promoter and were generated by the Transgenic RNAi Project at Harvard Medical School (Ni et al., 2008; Dietzl et al., 2007). They are shown in supplementary material Table S1. To express shRNA in the female germlines, fly lines expressing GAL4 under the maternal *α-tubulin67A* promoter (V37) and shRNA under UASp promoter were crossed and transheterozygous female progeny were examined. For observation of fixed mature oocytes, <1-day-old adult female flies were cultured with males on fresh food with dry yeast pellets for 3–5 days at 25°C. To overexpress BAF and BAF-3A, controlled under the UASp promoter in larval CNS, the GAL4 driver 167Y (Manseau et al., 1997) was used. *nhk-1^E60^* was examined over *Df(3R)ro80b*. Details of genes and mutations are as described previously (Lindsley and Zimm, 1992) and as in FlyBase (St. Pierre et al., 2014).

### Site-directed mutagenesis and molecular cloning of BAF-3A variants

DNA encoding BAF-3A (Lancaster et al., 2007) or BAF-3A (monomer) with a stop codon was cloned into pENTR/D-Topo (Invitrogen) and the missense mutations leading to the amino acid changes were introduced to BAF-3A by site-directed mutagenesis using the QuikChange kit (Agilent). After confirming the sequences, unmutated and mutated BAF-3A were transferred into pUASp using the Gateway vector pPWG and LR Clonase (Invitrogen), and used for P-element-mediated transformation of *w^1118^* by Genetic Service Inc. Five insertions were tested for each construct and gave similar results.

### Quantification of RNAi efficiency

Total RNA was purified using an RNAeasy kit (Qiagen) from 5–10 pairs of ovaries, which were dissected in Robb's medium from adult females matured for 4–8 days at 25°C. After genomic DNA was digested with DNase I (Qiagen), cDNA was generated using primers corresponding to target genes and Superscript III reverse transcriptase (Invitrogen). Real-time RT-PCR was performed in LightCycler 480 (Roche) using a pair of primers corresponding to target genes and SYBR Green Master mix (Roche). In parallel, a pair of primers corresponding to *actin 5C* gene were used as a control for normalisation.

### Cytological methods

Fluorescent *in situ* hybridisation of oocytes was carried out as described in Meireles et al. (Meireles et al., 2009) (except oocytes were fixed in 8% paraformaldehyde) using a fluorescently labelled probe generated by the following method. A 44-mer oligonucleotide, (CCCGTACTGGT)_4_, corresponding to the dodeca satellite (Abad et al., 1992) was incubated with terminal deoxynucleotidyl transferase (1.5 U/µl; Promega), Alexa-Fluor-546–dUTP (0.1 mM) and dTTP (0.8 mM) in transferase buffer (Promega) at 37°C for 1 hour and 70°C for 10 minutes, and purified through a G25 Mini Quick Spin Oligo Column (Roche). The chromosome arm length in wild-type was estimated as half of the distance between the Cen3 signals (2.16±0.84 µm). The arm length of undercondensed chromosomes in *Mi-2* RNAi was estimated by measuring the length of all visible chromosomes using the polygonal chain measurement tool in LSM Examiner (Zeiss) and dividing it by the number of visible arms for *Mi-2* RNAi (9.01±2.40 µm). The length of misaligned condensed chromosomes in *Mi-2* RNAi estimated by the above method (2.07±0.37 µm) was not statistically different from that of wild type. Mature oocytes were immunostained as described in Cullen and Ohkura (Cullen and Ohkura, 2001) using an anti-α-tubulin antibody (1∶250 DM1A; Sigma) and counterstained with DAPI (0.2 µg/µl; Sigma). The fixed oocytes were examined under PlanApochromat (63×, 1.4NA; Zeiss) using LSM510Exciter (Zeiss) attached to an Axiovert 200M (Zeiss). Typically, a series of *z*-sections were taken using 0.5-µm intervals. All images are shown as maximum intensity projections. Immunostaining of the squashed larval CNS was carried out using an anti-phospho-H3 (S10) antibody (1∶1000; 06-570; Upstate Biotechnology) and DAPI (0.2 µg/µl; Sigma) as described in Pimpinelli et al. (Pimpinelli et al., 2000) except that the dissected CNSs were fixed in 45% acetic acid for 30 seconds and 60% acetic acid for 30 seconds before squashing. For colchicine treatment, larval CNSs were incubated with 3 µg/ml colchicine in 0.7% sodium chloride for 30 minutes before hypotonic shock in 0.5% sodium citrate. The anti-phospho-H3 antibody faithfully highlighted mitotic cells in wild type and under BAF-3A overexpression, as all phospho-H3 positive cells show γ-tubulin accumulation at centrosomes (97/98 in wild type and 78/78 under BAF-3A overexpression). *nhk-1^E60^* might affect H3 phosphorylation, as some phospho-H3-positive cells (6/96) had no γ-tubulin accumulation at centrosomes. Images of fixed CNSs were captured under a PlanApochromat lens (63×, 1.4 NA; Zeiss) attached to an Axioplan 2 microscope (Zeiss) using a CCD camera (Orca; Hamamatsu) operated by OpenLab (Improvision).

For live imaging, oocytes from <1-day-old female flies matured with males at 18°C for 3–5 days were dissected in halocarbon oil (700, KMZ Chemicals) on a coverslip. The morphology of dorsal appendages was used for staging of oocytes, mature stage-14 oocytes were typically used unless stated otherwise. Images were taken using a PlanApochromat lens (63×, 1.4 NA; Zeiss) attached to an Axiovert 200M microscope (Zeiss) with a spinning disc confocal scan head (CSU-X1; Yokogawa) operated and analysed using Volocity (PerkinElmer). Typically a series of *z*-sections were taken covering the entire spindle volume using 0.8-µm intervals. All images are shown as maximum intensity projections. The contrast and brightness were adjusted uniformly across the entire field without changing, removing or adding features of images. The chromosome volume was measured using Volocity after the surface was determined automatically and adjusted by manually fine-tuning the pixel intensity threshold. Chi-square or Fisher's exact test and a modified Wilcoxon test were used for statistical analysis of categorical and parametrical data, respectively.

## Supplementary Material

Supplementary Material
