# Supplementary material for: The NuRD nucleosome remodelling complex and NHK-1 kinase are required for chromosome condensation in oocytes
Source: J Cell Sci. 2015 Feb 1;128(3):566–75. doi: 10.1242/jcs.158477 (PMC4311133; doi:10.1242/jcs.158477)
Supplement: Supplementary Material [file supp_128_3_566__index.html]

The NuRD nucleosome remodelling complex and NHK-1 kinase are required for chromosome condensation in oocytes — Supplementary Material 

# The NuRD nucleosome remodelling complex and NHK-1 kinase are required for chromosome condensation in oocytes

## JCS158477 Supplementary Material

**Files in this Data Supplement:**

- **Supplementary Material**
