# Supplementary material for: The NuRD nucleosome remodelling complex and NHK-1 kinase are required for chromosome condensation in oocytes
Source: J Cell Sci. 2015 Feb 1;128(3):566–75. doi: 10.1242/jcs.158477 (PMC4311133; doi:10.1242/jcs.158477)
Supplement: Supplementary Material [file supp_128.3.566_JCS158477.pdf]

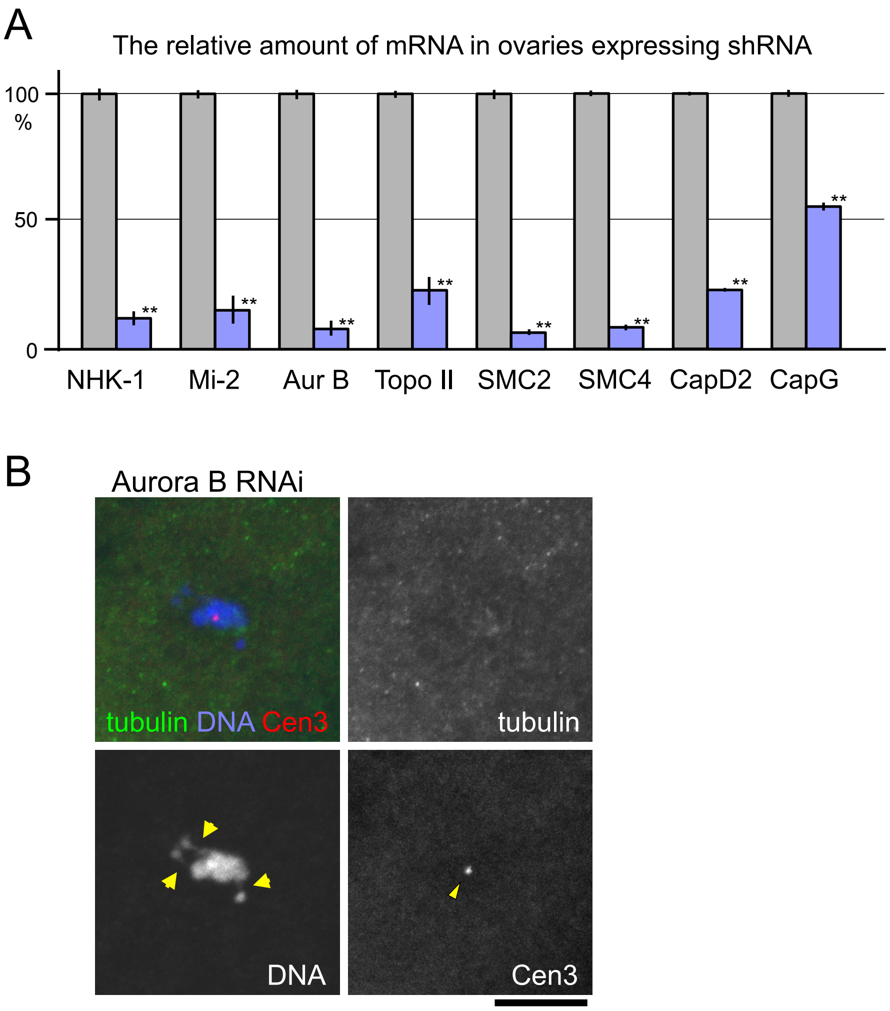

Figure S1. Depletion of mRNA in ovaries expressing shRNA, and the effects of *Aurora B* RNAi in chromosome condensation and microtubule assembly.
(A) mRNA levels of target genes greatly decreased in ovaries expressing shRNA. The relative amount of mRNA of *NHK-1*, *Mi-2*, *Aurora B*, *Topoisomerase II*, *SMC2, SMC4, CapD2* and *CapG* in ovaries expressing corresponding shRNA (blue bars) compared to ovaries expressing control shRNA (*white* gene; grey bars). Error bars represent standard errors of the mean (s.e.m) derived from biological triplicates (*NHK-1*, *Mi-2*, *Aurora B*, *Topoisomerase II*, *SMC2*) or qPCR triplicates (*SMC4, CapD2*, *CapG*). ** indicates significant differences from the control ($p<0.01$). (B) *Aurora B* RNAi in oocytes led to chromosome undercondensation and the absence of the spindle microtubules. Microtubules (tubulin; α-tubulin antibody), chromosome morphology (DNA; DAPI staining) and the positions of centromere 3 (Cen3; Dodeca satellite) in mature oocytes expressing *Aurora B* shRNA. Arrows and the arrowhead indicate thin DNA threads and Cen3 signal, respectively. Bar=10 μm.

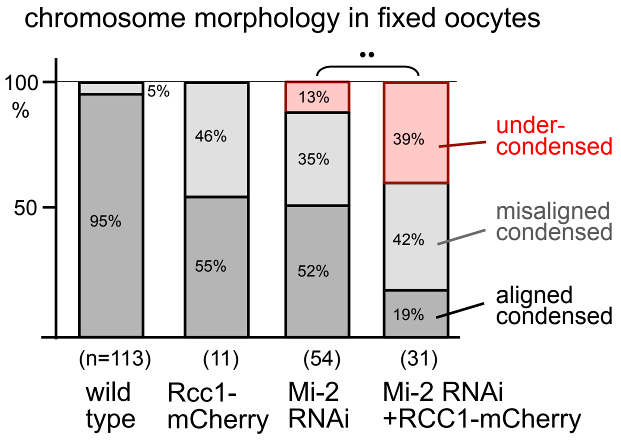

Figure S2. Expression of Rcc1-mCherry enhanced chromosome condensation defects in *Mi-2* RNAi oocytes.
The frequencies of chromosome morphology classes in DAPI-stained fixed mature wild-type oocytes or fixed mature oocytes expressing RCC1-mCherry alone, *Mi-2* shRNA alone or both *Mi-2* shRNA and Rcc1-mCherry. •• indicates a significant difference between with and without RCC1-mCherry expression ($p<0.01$) in terms of the frequency of undercondensed chromosomes induced by *Mi-2* RNAi.

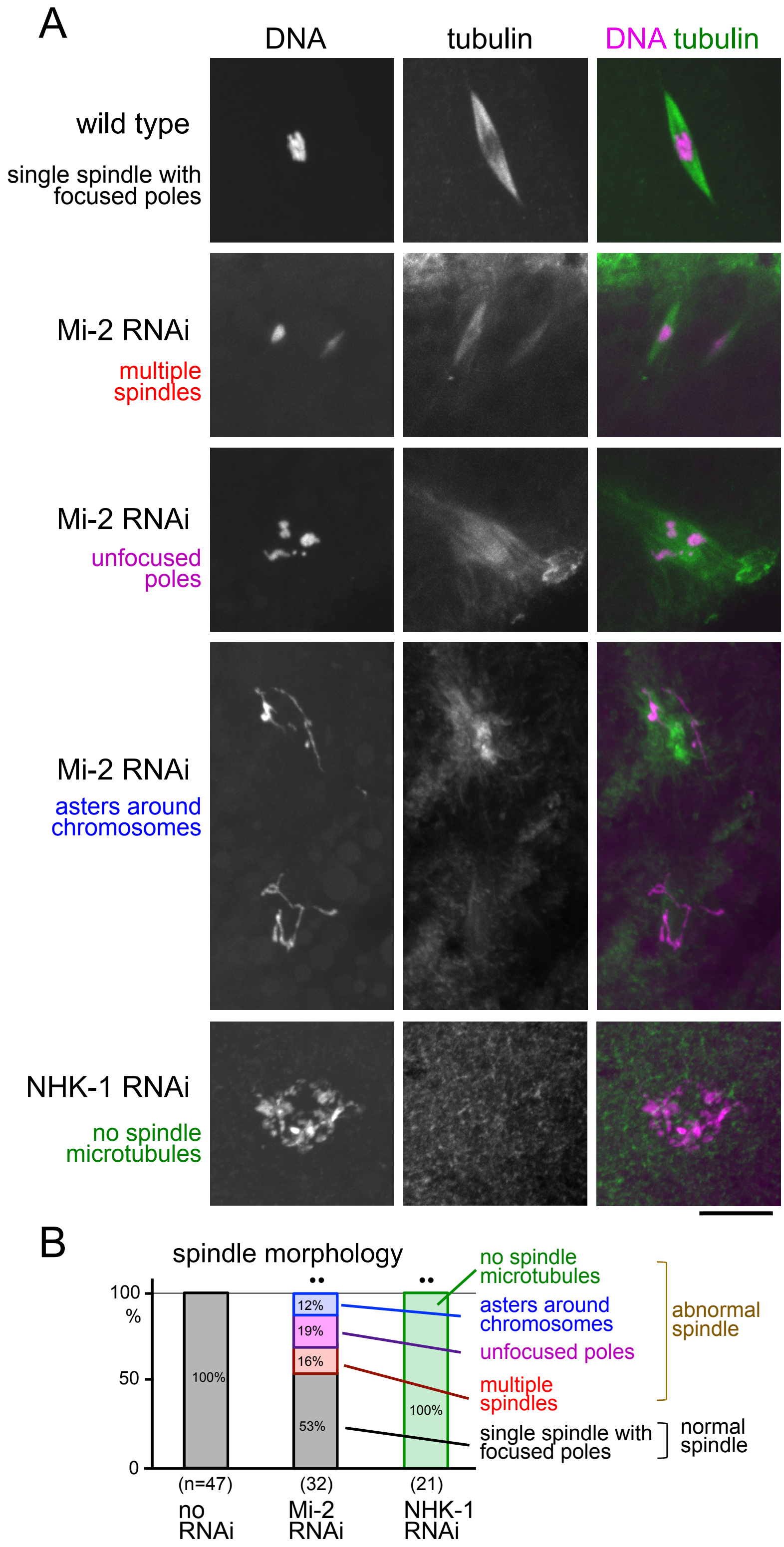

Figure S3. *Mi-2* and *NHK-1* RNAi disrupted bipolar spindle formation.
(A) Mature wild-type oocytes or oocytes expressing shRNA for *Mi-2* or *NHK-1* were immunostained for DNA and α-tubulin. Bar=10 μm. (B) The frequencies of the spindle morphology in wild-type mature oocytes or mature oocytes expressing shRNA for *Mi-2* or *NHK-1*. •• indicates a significant difference from wild type ($p<0.01$) in the frequency of abnormal spindles.

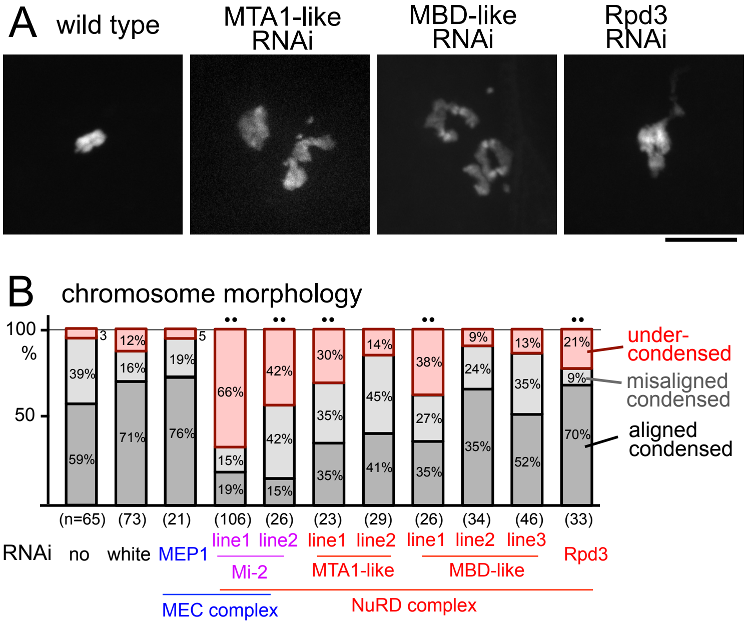

Figure S4. The NuRD complex is required for chromosome condensation.
(A) Chromosome morphology of mature oocytes expressing Rcc1-mCherry and shRNA for subunits of the NuRD complex or the MEC complex. Bar=10 μm. (B) The frequencies of chromosome morphology classes in wild-type oocytes, or oocytes expressing shRNA for *white*, *MEP-1*, *Mi-2* (line 1, 2), *MTA1-like* (line1, 2), *MBD-like* (line1, 2, 3) or *Rpd3*. •• indicates a significant difference from wild type ($p<0.01$) in terms of the frequency of undercondensed chromosomes. Multiple lines for *Mi-2*, *MTA1-like* and *MBD-like* express non-overlapping shRNA. Mi-2 is present in the MEC and NuRD complexes. MEP1 is specific to the MEC complex, whilst MTA1-like, MBD-like and Rpd3 are specific to the NuRD complex.

Table S1. RNAi lines used in this study

RNAi lines screened for chromosome condensation defects		
**Target gene**	**Target CG number**	**Fly line**
Arip4	CG4049	P{TRiP.HMS00584}attP2
Aurora B	CG6620	P{TRiP.GL00202}attP2
BAF	CG7380	P{TRiP.HMS00195}attP2
BEAF-32	CG10159	P{TRiP.GLV21006}attP2
Brm	CG5942	P{TRiP.HMS00050}attP2
Cap-D2	CG1911	P{TRiP.GL00523}attP2
Cap-D3	CG31989	P{TRiP.GL00575}attP2
Cap-G	CG34438	P{TRiP.GL00643}attP2
Cap-H	CG10726	P{TRiP.HMS00049}attP2
Cap-H2	CG14685	P{TRiP.GL00635}attP2
Chd1	CG3733	P{TRiP.GL00126}attP2
Chd3	CG9594	P{TRiP.HMS00302}attP2
CTCF	CG8591	P{TRiP.HMS02017}attP40
dom	CG9696	P{TRiP.HMS00142}attP2
dom	CG9696	P{TRiP.HMS02208}attP2
DREF	CG5838	P{TRiP.GL00532}attP2
dwg	CG2711	P{TRiP.GLV21031}attP2
Etl1	CG5899	P{TRiP.HMS00829}attP2
Hdac3	CG2128	P{TRiP.HMS00087}attP2
HDAC4	CG1770	P{TRiP.HMS00083}attP2
HDAC6	CG6170	P{TRiP.HMS00077}attP2
Hel25E	CG7269	P{TRiP.HMS00076}attP2
Hel89B	CG4261	P{TRiP.HMS00684}attP2
HP1b	CG7041	P{TRiP.HMS00396}attP2
Ino80	CG31212	P{TRiP.HMS00586}attP2
Iswi	CG8625	P{TRiP.HMS00628}attP2
Kis	CG3696	P{TRiP.HMS01254}attP2
Klp3A	CG8590	P{TRiP.HMS02192}attP40
Lds	CG2684	P{TRiP.HMS01389}attP2
Mcm2	CG7538	P{TRiP.HMS01520}attP2
MCPH1	CG42572	P{TRiP.HMS01688}attP40
Mi-2 ("line1")	CG8103	P{TRiP.GL00318}attP2
mor	CG18740	P{TRiP.GLV21027}attP2
NHK-1	CG6386	P{TRiP.GL00068}attP2
Okr	CG3736	P{TRiP.HMS00585}attP2
orc4	CG2917	P{TRiP.HMS00404}attP2
osa	CG7467	P{TRiP.HMS01738}attP40
Rm62	CG10279	P{TRiP.HMS00144}attP2
Rpd3	CG7471	P{TRiP.HMS00607}attP2
SAYP	CG12238	P{TRiP.HMS00337}attP2

Set8	CG3307	P{TRiP.GL00228}attP2
Sir2	CG5216	P{TRiP.HMS00484}attP2
Sirt2	CG5085	P{TRiP.GL01007}attP40
SMC2	CG10212	P{TRiP.GL00440}attP40
SMC4	CG11397	P{TRiP.GL00487}attP40
SNR1	CG1064	P{TRiP.HMS00363}attP2
Su(Hw)	CG8573	P{TRiP.HMS00970}attP2
Su(var)205	CG8409	P{TRiP.GL00531}attP40
Topoisomerase II	CG10223	P{TRiP.GL00338}attP2
Topors	CG15104	P{TRiP.HMS01149}attP2
Xnp	CG4548	P{TRiP.HMS00683}attP2
**Other RNAi lines used in this study**		
**Target gene**	**Target CG number**	**Fly line**
MBD-like (“line 1”)	CG8208	P{TRiP.HMS02825}attP40
MBD-like (“line 2”)	CG8208	P{TRiP.HMS01683}attP40
MBD-like (“line 3”)	CG8208	P{TRiP.GL00259}attP2
MEP-1	CG1244	P{TRiP.GL00319}attP2
Mi-2 (“line 2”)	CG8103	P{TRiP.HMC03329}attP40
MTA1-like (“line 1”)	CG2244	P{TRiP.HMS01251}attP2
MTA1-like (“line 2”)	CG2244	P{TRiP.HMS01084}attP2
white	CG2759	P{TRiP.GL00094}attP2
